# Identification of low-abundance proteins via fractionation of the urine proteome with weak anion exchange chromatography

**DOI:** 10.1186/1477-5956-9-17

**Published:** 2011-04-08

**Authors:** Chih-Ming Lu, Yu-Jen Wu, Cheng-Chi Chen, Jue-Liang Hsu, Jiing-Chuan Chen, Jeff Yi-Fu Chen, Chun-Hsiung Huang, Ying-Chin Ko

**Affiliations:** 1 Graduate Institute of Medicine, College of Medicine, Kaohsiung Medical University, Kaohsiung, Taiwan; 2 Department of Urology, Buddhist Da Lin Tzu Chi General Hospital, Chiayi, Taiwan; 3 Department of Beauty Science, Meiho University, Pingtung, Taiwan; 4 Graduate Institute of Biotechnology, National Pingtung University of Science and Technology, Pingtung, Taiwan; 5 Department of Food Science and Nutrition, Meiho University, Pingtung, Taiwan; 6 Department of Biotechnology, Kaohsiung Medical University, Kaohsiung, Taiwan; 7 Department of Urology, Center of Excellence for Environmental Medicine, Kaohsiung Medical University, Kaohsiung, Taiwan; 8 Center of Excellence for Environmental Medicine, Kaohsiung Medical University, Kaohsiung, Taiwan

**Keywords:** Weak anion exchange chromatography, DEAE-Sephacel, Fractionation, Proteomic, Urine

## Abstract

**Background:**

Low-abundance proteins are difficultly observed on the two-dimensional gel electrophoresis (2-DE) maps of urine proteome, because they are usually obscured by high-abundance proteins such as albumin and immunoglobulin. In this study, a novel fractionation method was developed for enriching low-abundance proteins by removing high-abundance proteins and progressive elution with salts of various concentrations.

**Results:**

Stepwise weak anion exchange (WAX) chromatography, which applied DEAE-Sephacel resin with non-fixed volume elution, was used to fractionate urine proteome prior to performing 2-DE. Urine proteome was separated into four fractions by progressively eluting the column with 0 M, 50 mM, 100 mM, and 1 M NaCl solutions. Most of the heavy and light immunoglobulin chains appeared in the eluent. After the high-abundance proteins were removed, various low-abundance proteins were enriched and could be easily identified. The potential of this method for obtaining diversified fractionations was demonstrated by eluting the column separately with Na_2_SO_4 _and MgCl_2 _solutions. The 2-DE maps of the fractions eluted with these different salt solutions of identical ionic strength revealed markedly different stain patterns.

**Conclusion:**

The present study demonstrated that this fractionation method could be applied for purposes of enriching low-abundance proteins and obtaining diversified fractionations of urine, and potentially other proteomes.

## Background

Two-dimensional gel electrophoresis (2-DE) is a powerful technique for resolving a complex protein mixture. The analysis of urine proteins by 2-DE offers the potential for diagnosing and monitoring the progression of various diseases [1-5]. For example, analyses of urine proteins for the identification of disease biomarkers have been applied in bladder cancer [6,7], lung cancer [8], ovarian cancer [9], prostate cancer [10], membranous nephropathy [11], diabetic nephropathy [12], nephritic syndrome [13], and glomerular nephrotoxicity [14]. Although several 2-DE maps of human urine have been published [15-17], the resolution of these maps remains insufficient s, and the demonstration of whole proteins in human urine remains a challenge. Immunoglobulin heavy and light chain proteins, as well as other high-abundance proteins, often obscure low-abundance proteins on 2-DE maps. An effective way to increase the resolution of urine proteome is to carry out a fractionation procedure prior to performing the 2-DE analyses. Certain fractionations have been widely used prior to 2-DE analysis in order to obtain more comprehensive information. For example, immunoaffinity subtraction chromatography [17], ligand beads [18], preparative electrophoresis and 2-DE [19], cation exchange chromatography in combination with a batch-absorption method [20], and finally, a commercially manufactured protein depletion kit to remove the six most abundant human plasma proteins (including albumin, transferrin, haptoglobin, immunoglobulin G, immunoglobulin A, and alpha-1 antitrypsin) [21] have all been utilized for this purpose.

There are many methods available for protein separation, and include, ammonium sulfate precipitation, gel-filtration, hydrophobic interaction chromatography, and ion exchange chromatography. Fractionation by ammonium sulfate precipitation depends on the solubility of protein. When the concentration (ionic strength) of the salt increases, solubility of the protein reduces. At a higher ionic strength, more proteins will be precipitated from the solution. Due to the inadequate protein resolution of ammonium sulfate precipitation, this method is usually only used in preliminary protein separation. While gel-filtration separates proteins based on the size of proteins, its main disadvantage is a limited loading capacity and low resolution associated with diffusion and turbulence. On the other hand, protein separation by hydrophobic interaction chromatography relies on differences in hydrophobic groups on the surface of solute. In this method, the hydrophobic groups of proteins bind the hydrophobic groups on the surface of an insoluble matrix. Further, ion exchange chromatography depends on charge-charge interaction between the fluid body proteins and the charges of the resin. In anion exchange chromatography, the binding ions have negative charge and the immobilized functional groups have positive charge. Once the solutes are bound to the gel, the column is washed with a starting buffer and the bound molecules are eluted off using a salt solution with various ionic strength. There are two main elution methods in chromatography: continuous gradient elution and stepwise isocratic elution. However, under equal volume of eluent, the best resolution can be obtained by using continuous gradient elution rather than stepwise isocratic elution. A low-abundance protein which is diluted in different fractions will reduce the possibility of a successful identification. To circumvent this problem, a stepwise, non-fixed volume, isocratic elution weak anion exchange (WAX) chromatography fractionation approach was investigated in this study. This modified WAX fractionation was comprised of four stepwise isocratic elusions. At each isocratic ionic strength, the elution process was continued until protein failed to be detected in the eluent. The aims of this study were to establish 2-DE maps with fine resolution and to enrich low-abundance proteins, thereby providing a platform for identification of potential disease biomarkers.

## Materials and methods

### Collection of urine

Urine samples were collected from 11 healthy males and 9 females between the ages of 18 and 54 years. Subjects with renal disorders or those being administered medication during the sample collection period were excluded. None of the females were menstruating at the time of sample collection. A 100 mL midstream urine sample was collected in the morning for each individual, and the samples were then combined together and supplemented with protease inhibitor cocktail to avoid proteolysis. Samples were centrifuged at 12,000 rpm at 4°C to remove cell debris and insoluble solids. The final supernatants (2L) were then loaded to a Stirred Ultrafiltration Cell 8400 (Millipore, Billerica, MA, USA) and YM5 membrane (5000 molecular weight cut-off) to concentrate the solution and remove small interference molecules. The concentrated urine samples had a final volume of 50 ml, and were stored at -80°C until further use. This protocol was approved by the Institute Review Board (Approval No. B09601020).

### Fractionation of urine proteome by non-fixed volume stepwise WAX

A column (5 cm × 10 cm) packed with 50 gram DEAE-Sephacel (a weak anion exchanger from GE Healthcare) was equilibrated five times with 50 mM Tris-HCl buffer prior to use. Twenty ml of concentrated urine samples were dialyzed overnight at 4°C with 50 mM Tris-HCl buffer (pH 8.0, 1 mM EDTA, 20 mM DTT). After removing the precipitate formed during dialysis by centrifugation (8000 rpm), the sample was added to the column. First, the column was eluted by 3000 ml of 50 mM Tris-HCl buffer without salt at a flow rate of 40 ml/hr until no protein was detected in the eluent by Bradford dye assay. This process is distinct from fixed volume elution, in which some proteins could present in more than one fraction. A total of 3000 ml of combined eluent was collected and concentrated to a volume of 50 ml by using a Stirred Ultrafiltration Cell 8400 and a YM5 membrane. This sample was referred to as "unbound". Next, a solution of 50 mM NaCl/50 mM Tris-HCl buffer was used to elute the column, again until no protein was detected in the eluent, and a total of 8000 ml solution was collected and concentrated to obtain the 2^nd ^fraction (referred to as "NaCl-1"). A total of 4500 ml of 100 mM NaCl/50 mM Tris-HCl buffer was collected for the next elution, followed by concentration to obtain fraction NaCl-2. A 4000 ml of 1 M NaCl/50 mM Tris-HCl buffer was collected for the last elution using to obtain fraction NaCl-3. The whole elution process for obtaining fractionations of urine protein was carried out in a 4°C cold chamber to maintain the stability of proteins.

### Protein precipitation by trichloroacetic acid/acetone

The urine protein mixture in the supernatant was precipitated out overnight at -20°C by triple the volume of 10% TCA/Acetone solution containing 20 mM DTT [3]. After centrifugation at 8000 rpm for 30 min at 4°C, the supernatant was discarded. The pellet was rinsed three times in cold acetone containing 20 mM DTT and air-dried, and subsequently resuspended overnight at 4°C in a rehydration buffer purchased from Bio-Rad (6 M urea, 2 M thiourea, 0.5% CHAPS, 0.5% IPG buffer, 20 mM DTT, 0.002% bromophenol blue). The protein contents were determined using a 2-D Quant Kit (GE Healthcare).

### Two-dimensional gel electrophoresis

The first dimension electrophoresis (isoelectric focusing) was performed on a Bio-Rad PROTEAN IEF Cell at 20°C with a current limit of 50 A per strip. A sample was dissolved in rehydration buffer (as described above) and applied on a IPG strip in a strip holder. Every 11-cm pI 4-7 IPG strip (Immobiline DryStrip) was rehydrated at 50 V for 12 h, then focused according to the preset program (200 V (1.5 h), 500 V (1 h), 1,000 V (1 h), 4,000 V (1 h), 8,000 V (2 h)) until the total Vh reached 19,960. On the other hand, every 11-cm pI 3-10NL IPG strip was rehydrated at 30 V for 12 h, then focused according to the preset program (200 V (1.5 h), 500 V (1 h), 1,000 V (1 h), 4,000 V (1 h), 8,000 V (3 h), until the total Vh reached 27,960.

After isoelectric focusing, the strip was removed and equilibrated for 10 min in a 5 ml buffer (50 mM Tris-HCl, pH 8.8, 6 M urea, 30% glycerol, 2% SDS, 0.01% bromophenol blue) containing 1% (w/v) DTT, then transferred to another 5 ml equilibration buffer containing 2.5% (w/v) iodoacetamide for an additional 10 min. The equilibrated strip was placed on the top of a SDS-PAGE gel (12.5%), sealed with 0.5% agarose, followed by the running of a second dimension electrophoresis at 150 V for 6.5 h. The electrophoretic unit used for the second dimension electrophoresis was an SE 600 Ruby (Hoeffer).

### Protein spot identification by LC-MS/MS

#### In-gel Digestion

A 1 mm × 1 mm piece of protein at the spot of interest was excised and placed into a microcentrifuge tube. A 100 μlof 50 mM DTT in 25 mM ammonium bicarbonate (pH 8.5) was added to the tube which was shaken for 1 h at 37°C. After removing excess DTT in the supernatant, 100 μl of 100 mM Iodoacetamide (IAA) in 25 mM ammonium bicarbonate (pH 8.5) was added to the tube which was shaken for 30 min at RT in the dark. The excess IAA in the supernatant was removed. Next, 100 μl of 50% acetonitrile in 25 mM ammonium bicarbonate buffer (pH 8.5) was added, and the gel piece was soaked for 15 minutes followed by a complete removal of the buffer. The destaining process was carried out two or more times depending on the intensity of the dye. The gel piece was soaked in a 100 μl of 100% acetonitrile for 5 minutes and dried by SpeedVac to remove the remaining acetonitrile. After drying, 0.1 μg of trypsin in 10 μl 25 mM ammonium bicarbonate (pH 8.5) was added to the gel piece. One hour later, another 100 μl 25 mM ammonium bicarbonate was added and digestion was run for 16 h at 37°C. Following, 50 μl of 5% TFA in 50% acetonitrile was added to quench the trypsin digestion. The solution was sonicated for 10 second to release the tryptic peptides from the gel. The peptide solution was concentrated for the following LC-MS/MS analysis.

#### LC-MS/MS analysis

The above peptide mixture was subjected to a CapLC system (Waters, Milford, MA,) using a capillary column (75 um i.d., 10 cm in length, C SUN, Taiwan) with a linear gradient from 5% to 50% acetonitrile containing 0.1% formic acid for a total duration of 46 minutes. The separated peptides were analyzed online under positive survey scan mode on a nano-ESI Q-TOF (Micromass, UK) instrument. The scan range was from m/z 400 to1600 for MS and m/z 50 to 2000 for MS/MS. The raw data was processed into a text file format of PKL with MassLynx 4.0 (subtract 30%, smooth 3/2 Savitzky Golay and center three channels 80% centroid).

#### Database search

For purposes of protein identification, the PKL files generated from MS/MS spectra were uploaded to the MASCOT search engine v2.2 (Matrix Science, UK) (http://www.matrixscience.com). The parameters selected for searching the database were as follows: a) protein database was set to NCBInr, b) taxonomy was set as human, c) a single trypsin missed cleavage was allowed, d) the mass tolerance was set to be 0.3 Da for both precursor and product ions, e) carbamidomethyl (C) was chosen as a fixed modification, f) deamidated (NQ), oxidation (M), Carbamidomethyl (K) and Carbamidomethyl (N-term) were chosen for variable modifications, and finally, g) the data was formatted into Micromass (.pkl) and ESI-QUAD-TOF was the chosen instrument. Proteins with scores above the significance threshold (p < 0.05) were identified. All significant hits have at least two matched peptides.

### Western blot analysis

Either 1-D or 2-D gel was transferred to a PVDF membrane (Millipore) for 1.5 h at 400 mA using Transphor TE 62 (Hoeffer). The membrane was then blocked overnight by a blocking solution (50 mM Tris-HCl, pH 8.0, 0.25% gelatin, 150 mM NaCl, 5 mM EDTA, 0.05% Tween 20). An antibody with appropriate dilution was added to the membrane and incubated at room temperature for 2 h. Rabbit anti-ZAG, anti-CRT, and anti-RBP4 polyclonal antibodies were purchased from ProteinTech Group, USA. Rabbit anti-HP polyclonal antibody was purchased from Rockland, USA. Mouse anti-b-actin monoclonal antibody was purchased from Sigma-Aldrich, USA. Mouse anti-Immunoglobulin monoclonal antibody was purchased from GenWay Biotech, USA. The membrane was washed three times in PBST (10 mM NaH_2_PO_4_, 130 mM NaCl, 0.05% Tween 20), then probed with the second Ab (goat anti-mouse IgG and horseradish peroxidase conjugate, 1:10,000 in blocking solution) for 1 h. After it was washed three times with PBST, the enzyme activity on the blot was visualized through chemiluminesence by adding ECL Western Blotting Reagents (Pierce).

## Results and Discussion

### 2-DE maps of urine proteome

A 2000 ml urine sample was collected from 20 healthy people. In order to eliminate individual differences, the samples were pooled together and concentrated by Stirred Ultrafiltration Cell 8400. The proteins were precipitated by 10% TCA/Acetone after dialysis.

**Figure 1:**
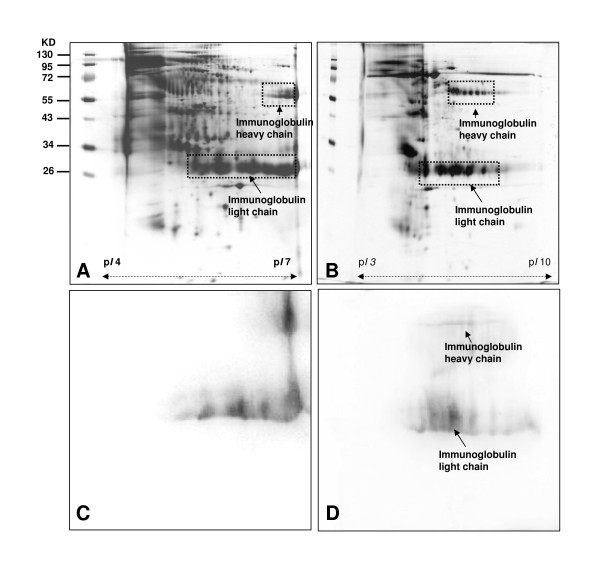
**Two-DE maps of urine proteome of healthy people**. (A) A total of 350 protein spots were detected at p*I *4-7, and (B) a total of 220 spots were detected at p*I *3-10NL by comparative analysis with PDQuest 2-D software. (C) 2-D western blot analysis immunoglobulin of total urine protein (p*I *4-7) (D) 2-D western blot analysis immunoglobulin of total urine protein (p*I *3-10NL).

An equal amount of 50 μg per gel was resolved in 2-DE using IPG strips (pI 4-7 and pI 3-10NL). The protein spots were visualized with silver stain and were excised for subsequent in-gel digestion and identification by LC-MS/MS. A total of 350 protein spots were detected in Figure 1A and a total of 220 spots were detected in Figure 1B by comparative analysis using PDQuest 2-D software (version 7.1.1). A direct 2-DE analysis of a complex protein sample can encounter resolution problems, and not all proteins can be detected on a 2-DE map. The difference in resolution could explain why more protein spots were detected in Figure 1A than in Figure 1B. The protein identification was carried out by LC-MS/MS. The spots N33 ~ N36, identified in Figures 1A and 1B, were immunoglobulin heavy and light chain proteins which were also listed in Table 1. Mouse anti-human immunoglobulin monoclonal antibody was also used for 2-D immunoreactivity analysis to confirm the data as shown in Figures 1C and 1D. The results clearly indicate that immunoglobulin heavy and light chain proteins are high-abundant in urine, which obscure the presence of the low-abundance proteins. Thus, it is necessary to remove the abundant proteins to enrich the low-abundance proteins. The application of nProtein A Sepharose (GE Healthcare) was widely used for removing immunoglobulin from a protein mixture. An attempt to remove immunoglobulin proteins in urine by nProtein A Sepharose was not successful (results reported in the additional file 1: Supplemental section and Additional file 2: figure S1).

**Table 1:** Summary of protein spots of healthy urine identified by LC-MS/MS

Spot number	Protein name	NCBI acc.no	Calculate Mr/p*I*	Peptide matched	Sequence covered %	MASCOT score
N1	Chain G, Gelsolin G4-G6 actin complex	1H1V_G	36.3/5.0	15	56	587
N2	Chain G, Gelsolin G4-G6 actin complex	1H1V_G	36.3/5.0	11	42	430
N3	keratin 10	XP_001169450	58.7/5.13	16	23	541
N4	Zn-alpha2-glycoprotein	CAA42438	34.7/5.71	10	20	249
N5	Zn-alpha2-glycoprotein	CAA42438	34.7/5.71	11	41	357
N6	Zn-alpha2-glycoprotein	CAA42438	34.7/5.71	11	30	317
N7	Zn-alpha2-glycoprotein	CAA42438	34.7/5.71	19	34	458
N8	mannose-binding lectin 2	NP_006807	40.2/6.46	8	9	187
N9	mannose-binding lectin 2	NP_006807	40.2/6.46	10	25	251
N10	mannose-binding lectin 2	NP_006807	40.2/6.46	13	23	262
N11	inter-alpha-trypsin inhibitor family heavy chain-related protein	BAA07602	103/6.51	10	9	453
N12	inter-alpha-trypsin inhibitor family heavy chain-related protein	BAA07602	103/6.51	18	11	456
N13	alpha-1-microglobulin/bikunin precursor	NP_001624	38.9/5.95	6	7	89
N14	alpha-1-microglobulin/bikunin precursor	NP_001624	38.9/5.95	11	28	308
N15	complex-forming glycoprotein HC	0801163A	20.4/5.84	24	50	348
N16	inter-alpha-trypsin inhibitor family heavy chain-related protein	BAA07602	103/6.51	7	8	261
N17	inter-alpha-trypsin inhibitor family heavy chain-related protein	BAA07602	103/6.51	12	11	286
N18	inter-alpha-trypsin inhibitor family heavy chain-related protein	BAA07602	103/6.51	8	7	296
N19	prostaglandin H2 D-isomerase	NP_000945	21.0/7.66	10	28	239
N20	heparan sulfate proteoglycan 2	XP_001099299	468/6.06	1	17	260
N21	heparan sulfate proteoglycan 2	XP_001099299	468/6.06	1	22	304
N22	mannan-binding lectin-associated serine protease-2	CAA67050	75.6/5.42	8	25	238
N23	mannan-binding lectin-associated serine protease-2	CAA67050	75.6/5.42	1	2	82
N24	retinol binding protein 4	1RBP	20.9/5.27	14	20	171
N25	retinol binding protein 4	1RBP	20.9/5.27	15	33	560
N26	immunoglobulin kappa light chain VLJ region	BAC01709	29.0/5.46	22	31	741
N27	immunoglobulin kappa light chain VLJ region	BAC01762	28.4/7.53	2	9	84
N28	immunoglobulin heavy chain	CAC10254	39.7/8.3	11	17	240
N29	immunoglobulin heavy chain	CAC10254	39.7/8.3	17	16	303
N30	vitelline membrane outer layer 1 isoform 1	NP_872372	21.5/4.9	3	24	99
N31	beta globin chain	AAA35952	18.9/6.28	2	13	99
N32	beta globin chain	ACF16774	11.4/6.9	5	48	192

### Fractionation of urine proteome by non-fixed volume stepwise WAX with NaCl solutions

**Figure 2:**
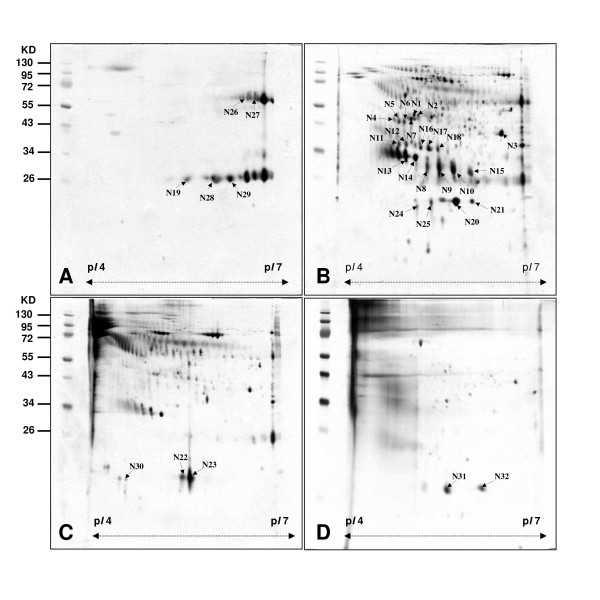
**2-DE maps of fractions of urine proteome of healthy people obtained by non-fixed volume stepwise elution DEAE-Sephacel anion exchange chromatography**. (p*I *4-7) (A) unbound Proteins in fraction (B) Proteins in fraction NaCl-1 obtained by elution with 50 mM NaCl. (C) Proteins in fraction NaCl-2 obtained by elution with 100 mM NaCl. (D) Proteins in fraction NaCl-3 obtained by elution with 1 M NaCl.

Ion exchange chromatography depends on charge-charge interactions between the proteins of a sample and the charges immobilized on the resin. In anion exchange chromatography the binding ions are negatively charged and the immobilized functional groups are positively charged. Once the solutes are bound to the gel, the column is washed in a starting buffer to equilibrate, then the bound molecules are eluted off in a stepwise manner using a salt solution with increasing ionic strength. At low ionic strengths, competition for charged proteins on gel resin is at a low level, therefore they are bound strongly on gel resin. When the ionic strength increases, so does the competition, and in turn, the affinity between proteins and gel resin decreases. The end result is more bound proteins being released from the gel resin. Thongboonkerd et al. used SP Sepharose Fast Flow Bead (a cationic ion exchange chromatography) to enrich the basic proteins in urine such as eosinophil-derived neurotoxin and interferon alpha [20]. This study used stepwise elution anion exchange chromatography for the fractionation of urine proteins and the enrichment of low-abundance proteins. The recovery rates of proteins were between 72.1% and 74.2%. The non-fixed volume stepwise elution method offers the potential of high resolution in preparative applications. Ideally, the non-fixed volume stepwise elution can eliminate the overlapping distribution of proteins in two neighboring fractions. The 2-DE maps of the four fractions of total urine protein eluted by NaCl (fraction Unbound, fraction NaCl-1, fraction NaCl-2, and fraction NaCl-3) were shown in Figure 2A to 2D.

**Figure 3:**
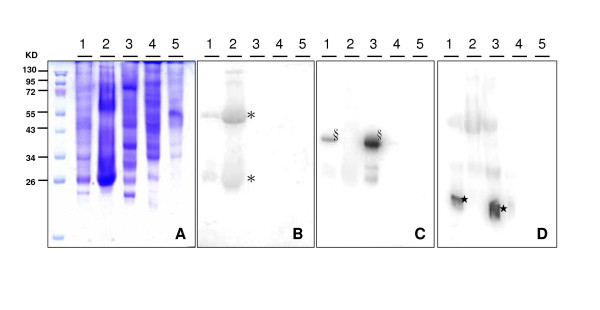
**Western blot analysis of immunoglobulins, zinc-alpha-2-glycoprotein and retinol-binding protein 4 in urine proteome and fractions of urine proteome of healthy people**. (A) CBR staining patterns of total urine protein (1), fraction Unbound (2), fraction NaCl-1 (3), fraction NaCl-2 (4) and fraction NaCl-3 (5). (B) Immunoreactivity to mouse anti-human immunoglobulin antibody. Two bands, indicating immunoglobulin heavy and light chain, appeared only in 2 (fraction Unbound) and total urine protein. (C) Immunoreactivity to mouse anti-human zinc-alpha-2-glycoprotein antibody. The band appeared only in 3 (fraction NaCl-1) and total urine protein. (D) Immunoreactivity to mouse anti-human retinol-binding protein 4 antibody. The band appeared only in 3 (fraction NaCl-1) and total urine protein.

The protein spots were visualized with silver stain which is a sensitive method for the appearance of protein with a limitation on dynamic range. In practice, the disadvantage for making more protein spots visible is the resulting smear around some spots. Three replicates were done for the 2D electrophoresis of each urine fractions. By comparative analysis using PDQuest 2-D software (version 7.1.1) a total of 40 ± 3.6 (average ± SD) protein spots (range 36-43) were detected in Figure 2A for fraction Unbound, a total of 278 ± 3.2 spots (range 274-280) were detected in Figure 2B for fraction NaCl-1, a total of 222 ± 8.3 spots (range 213-229) were detected in Figure 2C for fraction NaCl-2, and a total of 68 ± 5.6 spots (range 62-73) were detected in Figure 2D for fraction NaCl-3. Comparing with the 2-DE map of total urine protein without pre-fractionation, the number of spots recognized by PDQuest 2-D software was significantly higher. The additional file 1: Supplemental section and Additional file 3: figure S2A-S2D show the corresponding 2-DE maps of the fractions run by pI 3-10NL. The very different silver staining patterns of the four 2-DE maps indicate that an efficient fractionation of urine proteins was achieved. Most of the high-abundance proteins including immunoglobulin heavy and light chain proteins, appeared in Figure 2A. The low-abundance proteins were enriched in fraction NaCl-1 to fraction NaCl-3 (as shown in Figure 2B to 2D). The identities of 32 protein spots (N1 ~ N32) analyzed by LC-MS/MS after excision and in-gel digestion were reported in Table 1. The major protein spots identified in fraction Unbound were immunoglobulin heavy and light chain and prostaglandin H2 D-isomerase. When a mouse monoclonal antibody against human immunoglobulin was incubated in either total urine proteins versus the four fractions, immunoreactivities were present in total urine protein but only in fraction Unbound as shown in Figure 3B. In unison, it has been shown that the above fractionation method is effective in separating immunoglobulin heavy and light chain proteins from other urine proteins.

Some protein spots identified in fraction NaCl-1 and fraction NaCl-2 have several isoforms including, zn-alpha-2-glycoprotein (ZAG), inter-alpha-trypsin inhibitor family heavy chain-related protein (IHRP), retinol binding protein 4 (RBP4), heparan sulfate proteoglycan (HSPG), mannan-binding lectin-associated serine protease-2 (MASP-2), mannose binding lectin (MBL) and alpha-1-microglobulin/bikunin precursor (Figure 2B and 2C). In order to confirm the proteomic results, western blot analysis of some urine proteins was carried out. When a mouse monoclonal antibody of anti-human ZAG was incubated with total urine protein and the four fractions, immunoreactivities were identified in total urine protein but only in one of 4 fractions (fraction NaCl-1) as shown in Figure 3C. When a mouse antibody anti-human RBP4 was used, a similar phenomena was obtained as shown in Figure 3D. The results indicated that the stepwise elution DEAE-Sephacel anion exchange chromatography was effective in removing abundant proteins from total urine protein for the enrichment of low-abundance proteins.

Due to the presence of isoforms, only 15 proteins were identified from the 32 protein spots. It was likely that many of the proteins went through protein modification such as glycosylation or phosphorylation, presenting continuous spots on the 2-DE map. Each of the five proteins including ZAG, IHRP, RBP4, MASP-2, and HSPG with different p*I*, molecular weights, or isoforms appeared on the 2-DE map as more than one spot. ZAG (spot N4 ~ N7) is a glycoprotein with a molecular mass ~41 kDa. It appears in most body fluid, such as sweat, urine, blood, and cerebrospinal fluid. RBP4 (spot N24,25) is a carrier protein that transports retinol from the liver to the peripheral tissues and has been reported as a potential biomarker for glomerular disease [22]. As most reports of RBP4 in urine are related to disease, the significance of its existence in the urine of a healthy person is unknown. IHRP (spots N12, N16 ~ N18) is identified as an acute phase protein in animals that may function as an anti-inflammatory protein [23]. HSPG (spot N20, 21) have a potential role in glomerular filtration. Further, MBL (spot N8 ~ N10), a collagen-like serum protein, is a key component of innate immunity, which binds to carbohydrates on pathogens and mediates lectin-dependent activation of the complement pathway [24]. MASP-2 (spot N22, 23) is an enzyme of the innate immune system, which is activated when one of these proteins recognizes microorganisms and subsequently cleaves complement factors C4 and C2, and initiates the activation of the complement system [25]. All five proteins discussed above have direct links with immune response and inflammation, and have all been previously identified by Adachi et al. who used one-dimensional sodium dodecyl sulfate polyacrylamide gel and reverse phase high-performance liquid chromatography [26]. Although a large number of urine proteins were identified in this prior study (n = 1543), mass spectrometry analysis could not identify protein isoforms and possible post-translation modification. The strength of 2-DE analysis comes from both the qualitative and quantitative protein information revealed on the 2-DE maps. It is anticipated that potential disease biomarkers could be identified by detecting the qualitative and quantitative difference between the 2-DE maps of fractions of urine protein of patients and normal people using LC-MS/MS and western blotting analysis. In this study, DEAE-Sephacel was used as the gel resin in stepwise elution anion exchange chromatography with clean separation to fractionate total urine protein and enrich low-abundance proteins with encouraging results.

## Conclusions

The results demonstrated that non-fixed volume, stepwise elution, weak anion exchange chromatography with clean separation using DEAE-Sephacel as gel resin was an effective approach for the fractionation of total urine protein and the enrichment of low-abundance proteins. Four fractions of total urine protein were obtained by stepwise elution of the column, first without NaCl until no protein was detected in the eluting solution, followed by 50 mM NaCl solution until no protein was detected in the eluting solution. The next two fractions were obtained by eluting with 100 mM NaCl solution and 1 M NaCl solution, respectively. The 2-DE maps of the four fractions have been established. Identification of 32 protein spots by LC-MS/MS after excision and in-gel digestion have been carried out. Most of the immunoglobulin heavy and light chain proteins, which are abundant proteins in urine, only appeared in one of the fractions. Western blot analysis of immunoglobulin, ZAP, and RBP4 have been carried out to confirm the proteomic results. Identification of more protein spots are being carried out to better understand the whole scope of the urine proteins. This fractionation method is robust for the removal of high-abundance proteins, and can provide distinct fractionations for a more comprehensive profiling of protein expression. Although a large number of urine proteins could be identified by using the LC-MS/MS method alone, the MS analysis could not identify protein isoforms and possible post-translation modification. Overall, the non-fixed volume WAX fractionation coupled with the downstream 2-DE analysis can provide a more comprehensive display of differential proteins and is potentially useful for the identification and quantitation of protein isoforms and the detection of low-abundant proteins. Targeted MS screening approaches with sensitive detection and accurate quantification, such as multiple reaction monitoring (MRM), will be carried out to evaluate the differential proteins for the identification of potential disease biomarkers.

The solutions of two other salts, Na_2_SO_4 _and MgCl_2_, were also used for the elution. The method and result were reported in the additional file 1: Supplemental section and Additional file 4: figure S3. The 2-DE maps of the fractions obtained by elution with different salt solution possessing the same ionic strengths showed significantly different stain patterns. The results demonstrated the potential applications of this fractionation method using different salts for achieving diversified fractionation of human urine and enrichment of low-abundance proteins. It is believed that this method could be applied to the enrichment of low-abundance proteins in other body fluids such as serum, tear, saliva, cerebrospinal fluid, synovial fluid, cells, and tissues.

Wu et al. had reported a novel method for establishing a monoclonal antibody bank [27]. Applying complex antigens for mouse immunization and using 1-DE and 2-DE for immunoreactivity to identify monoclonal antibodies, a large number of pure monoclonal antibodies have been isolated. In this study, the developed stepwise elution DEAE-Sephacel anion exchange chromatography was effective in obtaining clean fractionations of urine proteins and enrichment of low-abundance proteins. A combination of both methods could offer a potentially promising way to establish a complete monoclonal antibody bank of urine proteome. Immunization with each fraction of urine proteins will be carried out to generate antibodies which will be separated into many pure monoclonal antibodies utilizing the method of Wu et al. The establishment of monoclonal antibody bank of urine proteome and the subsequent preparation of antibody chips of disease biomarkers could be used for simple and expedited disease screening.

## List of Abbreviations

2-DE: Two-dimensional gel electrophoresis; CRT: Calreticulin; Hp: Haptoglobin; HSPG: Heparan sulfate proteoglycan; IHRP: Inter-alpha-trypsin inhibitor family heavy chain-related protein; MASP-2: Mannan-binding lectin-associated serine protease-2; RBP4: Retinol-binding protein 4; WAX: Weak anion exchange; ZAG: Zinc-alpha-2-glycoprotein

## Competing interests

The authors declare that they have no competing interests.

## Authors' contributions

CL, YW, and CC collected the urine samples, performed the proteomic experiments, and wrote the manuscript. JH, JC, and JYC performed the proteomic experiments. CL, YW, CH, and YK designed the experiments and oversaw the IRB approval procedure. YK supervised the work, provided suggestions for solving problems, and revised the manuscript. All authors have read and approved the manuscript.

## Supplementary Material

Additional file 1 **Supplemental Section**. Three additional experiments: 1. Separation of immunoglobulin in urine proteome by nProtein A Sepharose, 2. 2-DE maps for pI 3-10, 3. Fractionation of urine proteome by non-fixed volume stepwise WAX with Na_2_SO_4_, and MgCl_2 _solutions. Click here for file

Additional file 2 **Figure S1**. Separation of immunoglobulin from urine of healthy people by nProtein A Sepharose. (A) Urine proteins not adsorbed to the gel resin. (B) Urine proteins adsorbed to the gel resin. The presence of immunoglobulin heavy and light chain proteins on both maps indicates that only partial removal of immunoglobulins was achieved by nProtein A Sepharose. Click here for file

Additional file 3 **Figure S2**. 2-DE maps of fractions of urine proteome of healthy people obtained by non-fixed volume stepwise elution DEAE-Sephacel anion exchange chromatography. (p*I *3-10NL) (A) Unbound proteins in fraction (B) Proteins in fraction NaCl-1 obtained by elution with 50 mM NaCl. (C) Proteins in fraction NaCl-2 obtained by elution with 100 mM NaCl. (D) Proteins in fraction NaCl-3 obtained by elution with 1 M NaCl. Click here for file

Additional file 4 **Figure S3**. Comparison of 2-DE maps of fractions of urine proteome of healthy people obtained by non-fixed volume stepwise elution DEAE-Sephacel anion exchange chromatography. (p*I *3-10NL) (A) Proteins in fraction NaCl-1 obtained by elution with 50 Click here for file
