# Supplementary material for: Identification of low-abundance proteins via fractionation of the urine proteome with weak anion exchange chromatography
Source: Proteome Sci. 2011 Apr 8;9:17. doi: 10.1186/1477-5956-9-17 (PMC3079594; doi:10.1186/1477-5956-9-17)
Supplement: Additional file 1 — Supplemental Section. Three additional experiments: 1. Separation of immunoglobulin in urine proteome by nProtein A Sepharose, 2. 2-DE maps for pI 3-10, 3. Fractionation of urine proteome by non-fixed volume stepwise WAX with Na2SO4, and MgCl2 solutions. [file 1477-5956-9-17-S1.DOC]

**Supplemental Section**

## Materials and methods

**Separation of immunoglobulin in urine proteome by nProtein A Sepharose**

A column (1cm × 3cm ) packed with 2.4 gram of nProtein A Sepharose was equilibrated with start buffer (50 mM boric acid, 50 mM Na_2_PO_4_, 50 mM Na citrate, 4.0 M NaCl, pH 9.0). A 2 ml of concentrated total urine protein was passed through the column along with a start buffer at a flow rate of 40 ml/hr. Subsequently, 15 ml of eluent was collected as fraction A-1. The proteins adsorbed on column were washed out using elution buffer (50 mM boric acid, 50 mM Na2PO4, 50 mM Na citrate, pH 3.0) and 10 ml of eluent was collected as fraction B-1. Fraction A-1 was passed through the column one more time. The eluent was collected as fraction A-2. The proteins absorbed on column were washed out with 10 ml elution buffer to obtain fraction B-2. The process was repeated one more time to obtain fraction A-3 and fraction B-3. Fraction A-3 contained the proteins not adsorbed on the gel resin. Fraction B-1, fraction B-2, and fraction B-3 were combined as fraction B-4 which contained the proteins adsorbed on the gel resin. Fraction A-3 was precipitated out overnight (-20°C) by triple the volume of 10% TCA/Acetone solution containing 20 mM DTT, then run 2-DE. Fraction B-4 was treated by the same procedures, then run 2-DE.

**Fractionation of urine proteome by non-fixed volume stepwise WAX with Na_2_SO_4_, and MgCl_2_ solutions**

A column (5cm × 10 cm) packed with 50 gram DEAE-Sephacel (a weak anion exchanger from GE Healthcare) was equilibrated five times with 50 mM Tris-HCl buffer before use. Samples containing 20 ml of concentrated urine were dialyzed overnight at 4°C with 50 mM Tris-HCl buffer (pH 8.0, 1 mM EDTA, 20 mM DTT). After the removal of the precipitate formed during dialysis by centrifugation (8000 rpm), the sample was added to the column. First, the column was eluted by 3000 ml of 50 mM Tris-HCl buffer without salt at a flow rate of 40 ml/hr. The column was eluted until no protein was detected in the eluent by Bradford dye assay. This process is very different from the fixed volume elution, in which some proteins could present in more than one fraction. A total of 6000 ml combined eluent was collected and concentrated by Stirred Ultrafiltration Cell 8400 and YM5 membrane to a volume of 50ml. The sample was called fraction Unbound. Then, a solution of 50 mM NaCl/50 mM Tris-HCl buffer was used to elute the column until no protein was detected in the eluent and a total of 8000 ml solution was collected and concentrated by the same procedure to obtain the fraction NaCl-1. A total of 4500 ml of 100 mM NaCl/50 mM Tris-HCl buffer was collected for the next elution until no protein was detected in the eluent, followed by concentration to obtain fraction NaCl-2. A 4000 ml of 1M NaCl/50 mM Tris-HCl buffer was collected for the last elution using the same procedure to obtain fraction NaCl-3. When Na_2_SO_4_ or MgCl_2_ were used to instead of NaCl in the elution, in order to have the same ionic strength as NaCl solution, the corresponding eluting salt concentrations were 16.7 mM, 33.3 mM, and 0.33 M, respectively (table S1). The fractions obtained after elution with Na_2_SO_4_ solutions were named fraction Na_2_SO_4_-1, fraction Na_2_SO_4_-2, and fraction Na_2_SO_4_-3. The fractions obtained after elution with MgCl_2_ solutions were named fraction MgCl_2_-1, fraction MgCl_2_ -2, and fraction MgCl_2_ -3 respectively.

## Results and Discussion

**Removal of immunoglobulin in total urine proteins by nProtein A Sepharose**

It is necessary to remove the high-abundant proteins to enrich the low-abundant proteins. nProtein A Sepharose (GE Healthcare) was widely used for removing immunoglobulin from a protein mixture. It was anticipated that the immunoglobulin proteins in urine could be removed by nProtein A Sepharose. According to the description in materials and methods section, fraction B-4 contained the proteins adsorbed on the gel resin. Fraction A-3 contained the proteins not adsorbed on the gel resin. The 2-DE map of fraction A-3 was shown in Additional file 1, Figure S1A and the 2-DE map of fraction B-4 was shown in Additional file 1, Figure S1B. The presence of both immunoglobulin heavy and light chain proteins on both maps indicated that only partial removal of immunoglobulin was achieved by nProtein A Sepharose. Additional file 1, Figure S1B showed that some other urine proteins also adsorbed by nProtein A Sepharose along with immunoglobulin. An alternative method would be investigated to solve the problem of incomplete immunoglobulin removal along with possible removal of some significant low-abundant proteins

**Fractionation of urine proteome of healthy people by non-fixed volume stepwise WAX with Na_2_SO_4_, and MgCl_2_ solutions**

The total urine protein had been fractionated effectively by stepwise elution using NaCl solution with increasing concentration. Different salts have different charge-charge interactions with the immobilized charges on the resin and the bound proteins. It was anticipated that different fractionations could be obtained by eluting the column with different salt solutions and the result could be a diversified fractionation of total urine protein. This could offer a diversified way to enrich more low-abundant proteins for the establishment of a complete 2-DE map of total urine protein. A divalent anion salt, Na_2_SO_4_ and A divalent cation salt, MgCl_2_ were chosen for the experiment. The goal was to use different salt solution with the same ionic strength for a comparison. Concentrations of NaCl, , Na_2_SO_4_ and MgCl_2_ with corresponding ionic strength are shown in Table S1.

When Na_2_SO_4_ or MgCl_2_ was used instead of NaCl as the eluting salt, the corresponding eluting salt concentration were 16.7 mM, 33.3 mM, and 0.33 M respectively. The fractions obtained after elution with MgCl_2_ solutions were named fraction MgCl_2_-1, fraction MgCl_2_-2, and fraction MgCl_2_-3, and their 2-DE maps were shown in Additional file 1, Figure S3D to S3F. The distinct stain patterns indicated that an effective fractionation was achieved. The fractions obtained after elution with Na_2_SO_4_ solutions were named fraction Na_2_SO_4_-1, fraction Na_2_SO_4_-2, and fraction Na_2_SO_4_-3, and their 2-DE maps were shown in Additional file 1, Figure S3G to S3I. The 2-DE maps of fraction NaCl-1, fraction NaCl-2, and fraction NaCl-3 reappeared in Figure 3A-3C for a better comparison between the 2-DE maps of fractions obtained by elution with different salt solutions possessing the same ionic strengths. For each of the three different ionic strengths (0.05, 0.1, and 1), the 2-DE maps of the fractions obtained by using NaCl, Na_2_SO_4_ , and MgCl_2_ eluting solutions were very different from each other. Identification of the protein spots will be carried out. This encouraging result demonstrated the potential application of this fractionation method for urine and other proteomes to obtain diversified fractions using various salts with different concentrations.

Table S1. Three ionic strengths with corresponding concentrations of NaCl, Na_2_SO_4_, and MgCl_2_ solutions

| Ionic strength | 0.05 | 0.1 | 1 |
| --- | --- | --- | --- |
| Conc. of NaCl | 50 mM | 100 mM | 1000 mM |
| Conc. of Na_2_SO_4_ | 16.7 mM | 33.3 mM | 330 mM |
| Conc. of MgCl_2_ | 16.7 mM | 33.3 mM | 330 mM |

**Figure S1.** Separation of immunoglobulin from urine of healthy people by nProtein A Sepharose. (A) Urine proteins not adsorbed to the gel resin. (B) Urine proteins adsorbed to the gel resin. The presence of immunoglobulin heavy and light chain proteins on both maps indicates that only partial removal of immunoglobulins was achieved by nProtein A Sepharose.

**Figure S2.** 2-DE maps of fractions of urine proteome of healthy people obtained by non-fixed volume stepwise elution DEAE-Sephacel anion exchange chromatography. (p*I* 3-10NL) (A) Unbound proteins in fraction (B) Proteins in fraction NaCl-1 obtained by elution with 50 mM NaCl. (C) Proteins in fraction NaCl-2 obtained by elution with 100 mM NaCl. (D) Proteins in fraction NaCl-3 obtained by elution with 1 M NaCl.

**Figure S3.** Comparison of 2-DE maps of fractions of urine proteome of healthy people obtained by non-fixed volume stepwise elution DEAE-Sephacel anion exchange chromatography. (p*I* 3-10NL) (A) Proteins in fraction NaCl-1 obtained by elution with 50 mM NaCl. (B) Proteins in fraction NaCl-2 obtained by elution with 100 mM NaCl. (C) Proteins in fraction NaCl-3 obtained by elution with 1 M NaCl. (D) fraction MgCl_2_-1 obtained by elution with 16.7 mM MgCl_2_ (E) Proteins in fraction MgCl_2_-2 obtained by elution with 16.7 mM MgCl_2_ (F) Proteins in fraction MgCl_2_-3 obtained by elution with 0.33 M MgCl_2_ (G) Proteins in fraction Na_2_SO_4_-1 obtained by elution with 16.7 mM Na_2_SO_4_ (H) Proteins in fraction Na_2_SO_4_-2 obtained by elution with 33.3 mM Na_2_SO_4_ (I) Proteins in fraction Na_2_SO_4_-3 obtained by elution with 0.33 M Na_2_SO_4_
