# Supplementary figures and images for: Identification of low-abundance proteins via fractionation of the urine proteome with weak anion exchange chromatography
Source: Proteome Sci. 2011 Apr 8;9:17. doi: 10.1186/1477-5956-9-17 (PMC3079594; doi:10.1186/1477-5956-9-17)

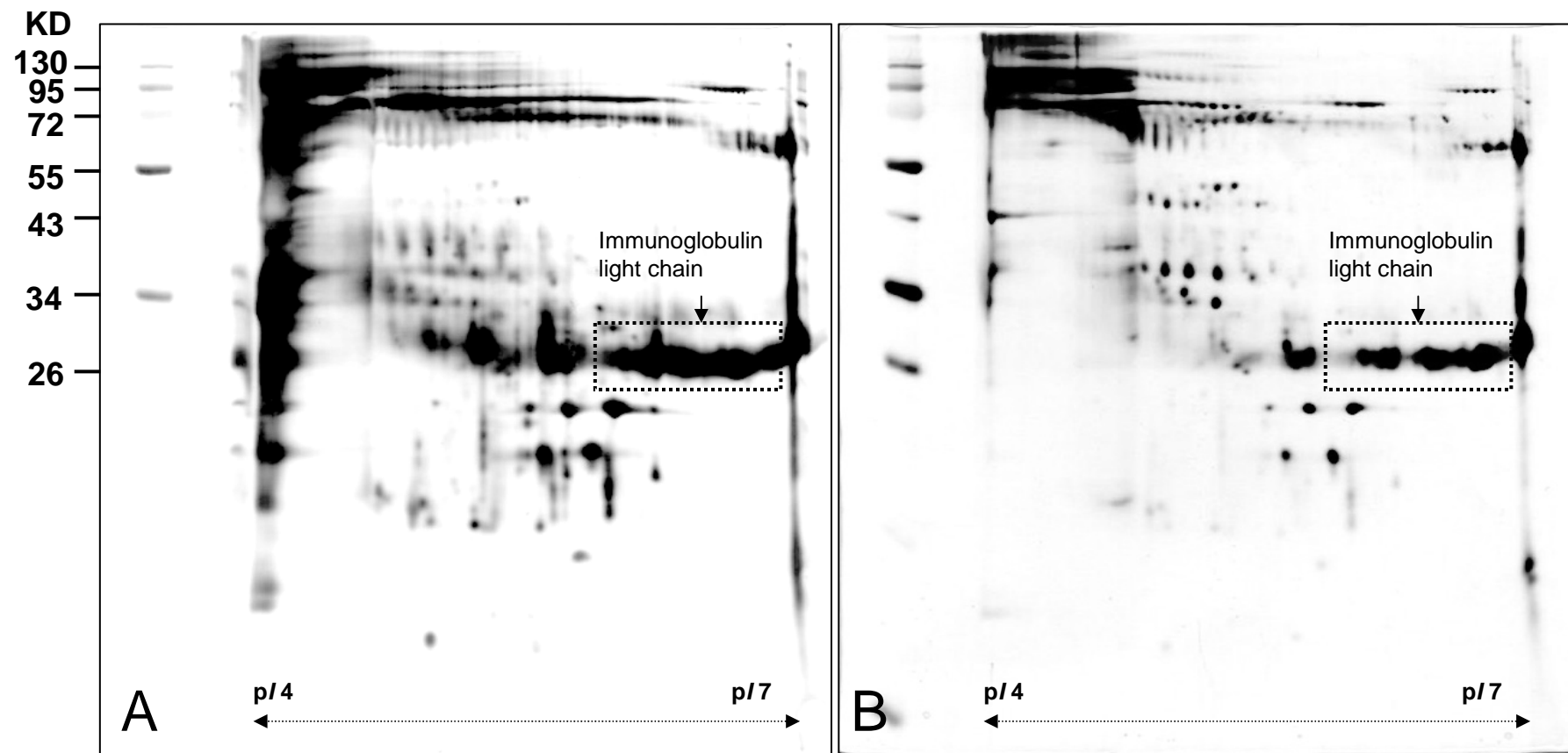

Supplement: Additional file 2 — Figure S1. Separation of immunoglobulin from urine of healthy people by nProtein A Sepharose. (A) Urine proteins not adsorbed to the gel resin. (B) Urine proteins adsorbed to the gel resin. The presence of immunoglobulin heavy and light chain proteins on both maps indicates that only partial removal of immunoglobulins was achieved by nProtein A Sepharose. [file 1477-5956-9-17-S2.PDF]

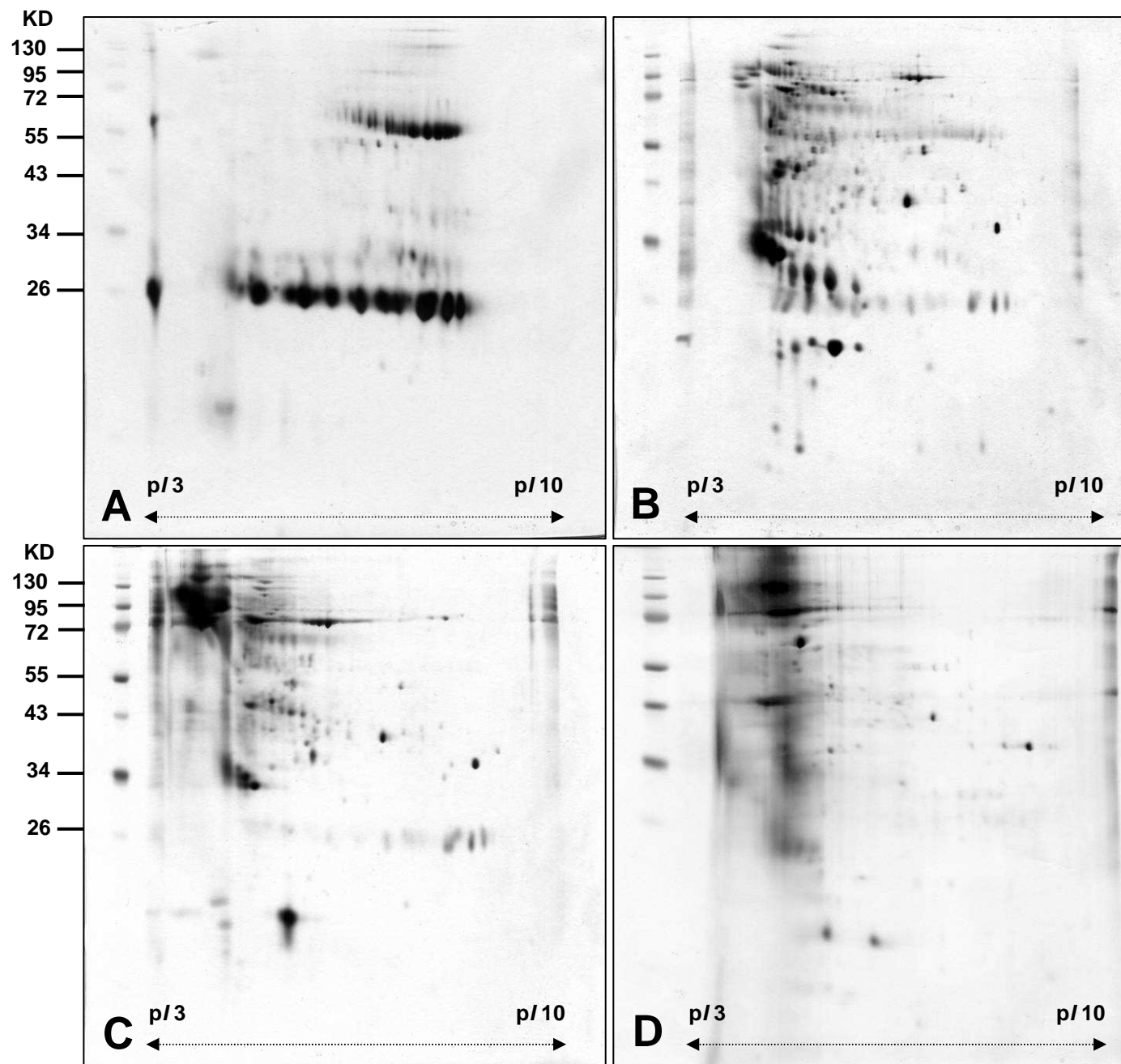

Supplement: Additional file 3 — Figure S2. 2-DE maps of fractions of urine proteome of healthy people obtained by non-fixed volume stepwise elution DEAE-Sephacel anion exchange chromatography. (pI 3-10NL) (A) Unbound proteins in fraction (B) Proteins in fraction NaCl-1 obtained by elution with 50 mM NaCl. (C) Proteins in fraction NaCl-2 obtained by elution with 100 mM NaCl. (D) Proteins in fraction NaCl-3 obtained by elution with 1 M NaCl. [file 1477-5956-9-17-S3.PDF]

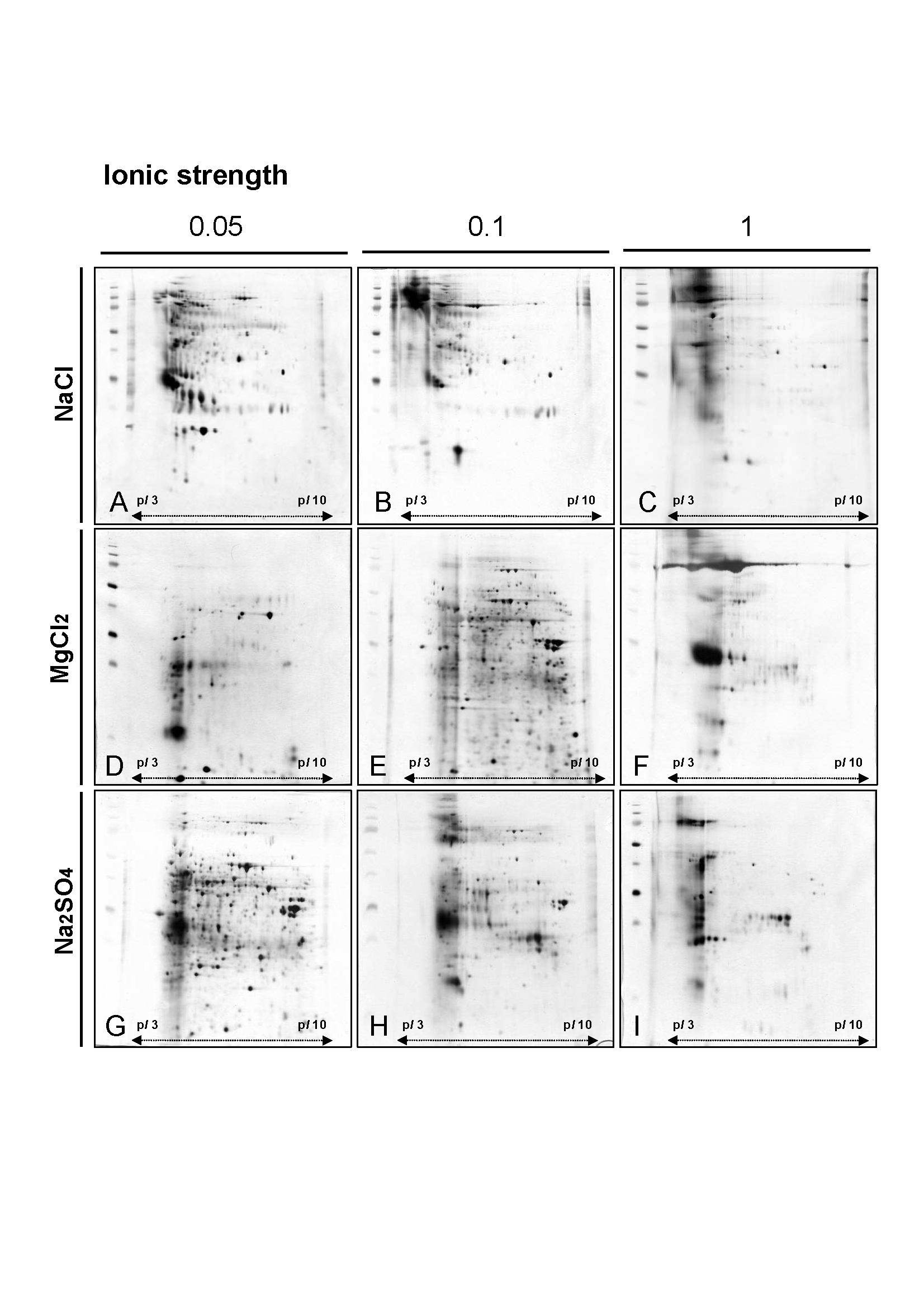

Supplement: Additional file 4 — Figure S3. Comparison of 2-DE maps of fractions of urine proteome of healthy people obtained by non-fixed volume stepwise elution DEAE-Sephacel anion exchange chromatography. (pI 3-10NL) (A) Proteins in fraction NaCl-1 obtained by elution with 50 [file 1477-5956-9-17-S4.TIFF]
